# Mangrove roots model suggest an optimal porosity to prevent erosion

**DOI:** 10.1038/s41598-021-88119-5

**Published:** 2021-05-11

**Authors:** Amirkhosro Kazemi, Luciano Castillo, Oscar M. Curet

**Affiliations:** 1 Ocean and Mechanical Engineering, Florida Atlantic University, Boca Raton, 33431 USA; 2 School of Mechanical Engineering, Purdue University, West Lafayette, 47907 USA; 3 Ocean and Mechanical Engineering, Florida Atlantic University, Boca Raton, 33431 USA

**Keywords:** Fluid dynamics, Marine biology, Physical oceanography

## Abstract

Mangrove swamps are extremely productive ecosystems providing many ecological services in coastal regions. The hydrodynamic interactions of mangrove roots and water flow have been proposed as a key element to mitigate erosion. Several studies reveal that precise prediction of the morphological evolution of coastal areas, in the face of global warming and the consequent sea-level rise, requires an understanding of interactions between root porosity (the fraction of the volume of void space over the total volume), water flows, and sediment transport. Water flows around the mangrove prop roots create a complex energetic process that mixes up sediments and generates a depositional region posterior to the roots. In this work, we investigated the boundary layer behind permeable arrays of cylinders (patch) that represent the mangrove roots to explore the impact of patch porosity on the onset of sediment transport. The flow measurements were performed in a vertical plane along the water depth downstream of the mangrove root models. A high-resolution Particle Image Velocimetry (PIV) was used in a flume to observe the impact of porosity on the mean flow, velocity derivatives, skin friction coefficient, and production of turbulent kinetic energy for Reynolds number of 2500 (based on patch diameter length-scale). Here, we proposed a predictive model for critical velocity for incipient motion that takes into account the mangrove roots porosity and the near-bed turbulence effect. It is found that the patch with the $\phi =47\%$ porosity, has the maximum critical velocity over which the sediment transport initiates. We found the optimum porosity has the minimum sediment erosion and creates negative vorticity sources near the bed that increases the critical velocity. This signifies an optimum porosity for the onset of sediment transport consistent with the porosity of mangroves in nature. The phenomenological model is elucidated based on an analysis of the vorticity evolution equation for viscous incompressible flows. For the optimum porous patch, a sink of vorticity was formed which yielded to lower the near-bed turbulence and vorticity. The minimum velocity fluctuations were sufficient to initiate the boundary layer transition, however, the viscous dissipation dominated the turbulence production to obstruct the sediment transport. This work identified the pivotal role of mangrove root porosity in sediment transport in terms of velocity and its derivatives in wall-bounded flows. Our work also provides insight into the sediment transport and erosion processes that govern the evolution of the shapes of shorelines.

## Introduction

While global climate change is presently creating several environmental concerns, one of the most well-publicized challenges is the expected rise in sea levels^1^. Recent projections of expected sea-level rise by 2100 could reach around 30–60 cm by 2100 even if greenhouse gas emissions are sharply reduced and global warming is limited to well below 2 °C, but around 60–110 cm if greenhouse gas emissions continue to increase strongly^2^. The ramifications of increasing coastal flooding worldwide thus necessitate the development of novel solutions and improved buffer mechanisms^3^. Biomimicry design of an artificial mangrove system that replicates the protective effects observed in nature can mitigate the effect of coastal flooding and adapt the low-lying coastal areas to a world with higher oceans. Understanding the mechanism of erosion and sedimentation in mangrove swamp is a fundamental key in coastal and river management, both to protect ecological services and control flood and erosion risks. In addition, the understanding of the fluid dynamics around the prop roots is expected to a critical aspect of designing a resilient ocean structure to mitigate erosion.

**Figure 1:**
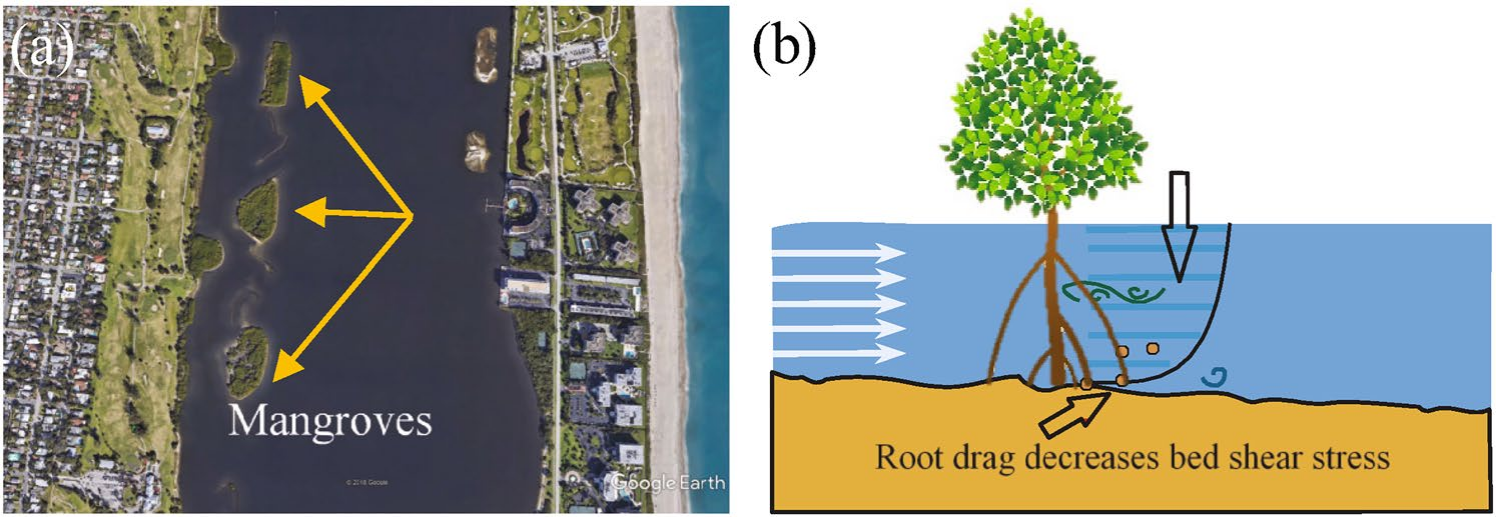
(**a**) Emergent patches of mangroves in Lake Worth Lagoon, Florida provide nursery habitat for species and animals by accumulating sediments and impede erosion due to the dense root structure. Google earth Pro 7.3.3.7786 (August 28, 2020). Lake Worth Lagoon, Florida. 26$^\circ$ 37′ 42.09″ N, 80$^\circ$ 02′ 34.27″ W, Eye alt 2200 m https://www.google.com/earth/. (**b**) The elevated drag force dominated by dense roots creates a small eddy size and affects bed shear stress. The top arrow and the lines represent the vortex shedding and velocity profile, respectively, which enhances the sediment trap by modulating the near-bed flow structure.

Mangrove vegetation naturally grows in shorelines of (sub) tropical regions and influences the development of the intercoastal areas^4–7^ (Fig. 1a). Mangrove vegetation provides a wide range of ecological ecosystem functions such as reducing coastal erosion, promoting biodiversity, removal of nitrogen and phosphorus and carbon dioxide sequestrations^3,8–13^. Mangrove species have various complex root systems. Avicennia have lenticel-equipped pneumatophores (upward-directed roots) that oxygen passively diffuses through roots^9^. The lenticels can be closed, partially opened, or fully opened, depending on environmental conditions^14^. The spongy pneumatophores are normally short (less than 30 cm), however, grow much larger and become more numerous in Avicennia marina living in anaerobic and oil-polluted conditions^9^. Oxygen can also penetrate through non-lenticellular portions of the pneumatophores^15^. Prop roots are the aerial roots of Rhizophora mangle, as many of them living in the Indian River Lagoon, Florida^16^ and Rookery Bay, Florida^17^. The submerged prop roots provide solid surfaces for attachment of a variety of marine invertebrates^9^. In addition, many fish species use the habitat as a nursery area; the complex tangle of roots provides an ideal refuge from predators^9^. Mangroves inhabit only around 0.5% of global coastal ocean area, yet they capture carbon dioxide ($\text{CO}_2$) from the atmosphere through complex biological processes that comprise almost 10–15% of total carbon sequestration^18–20^. They most likely store up to 20 Pg C, equivalent to nearly 2.5 times annual global $\text{CO}_2$ emissions^21^. In Puerto Rico, Golley et al.^22^ explored partitioning of the total biomass of a mangrove tree and observed that peat and fine roots and the accumulation of organic material as peat in mangrove soils could serve as a sink for carbon, and nitrogen. Mangroves that inhabit soft-sediment deposits, can capture carbon passively and actively from water currents discharged from upstream and from tidal water from the coastal ocean^19^.

These vital ecological functions are influenced by the flow around the intricate prop roots^23^. Rhizophora prop roots protrude in shorelines, extend from the tree trunk and form a radial, circular patch or large-scale mangrove forests. The roots enhance the mangrove drag and in tidal currents flows the frictional effect due to the drag can cause the currents to rotate into the root patch. The roots generate small-scale turbulence through the developments of vortex shedding and eddy formation^24,25,26^ (Fig. 1b). The trunk, branches, leaves, and roots of the mangrove act as an obstruction to the water flow adding a biological dimension to the complex interactions between hydrodynamics and sediment movement in coastal areas^27–31^.

The water current velocities in mangrove sites mainly depend on the creek depth and the site location. In Everglades National Park, Florida, velocities of less than 20 $\frac{\rm{cm}}{\rm{s}}$ were observed in the red mangrove areas at about 1 m water height above the bed^10^. Horstman et al.^32^ conducted field measurements at the north section of Mae Nam Trang Creek in the Kantang estuary in Trang province in Thailand and discovered that inside the mangrove swamps water flowed nearly parallel to the corresponding mangrove swamp for shallow water depths ranging from 7 to 150 cm^32^. Similarly, Wolanski^33^ performed field observations of water current within immensely vegetated swamps and reported that peak tidal velocities at 50 m away from Coral Creek in Australia were less than 7 $\frac{\rm{cm}}{\rm{s}}$ in shallow water under 1 m depth. Katherisan^34^ observed water flow in the Vellar Estuary on the southeastern coast of India and reported that tidal velocities within the mangrove vegetation were approximately 9 $\frac{\rm{cm}}{\rm{s}}$ compared to no mangrove bank areas where the velocities were nearly between 18 and 20 $\frac{\rm{cm}}{\rm{s}}$ in 1.5 m water depth.

The mangrove prop roots also affect flow structure and turbulence with a subsequent impact on the onset of sediment transport^35^. The mangrove roots disturb velocity and turbulence intensity profiles near the riverbed that deviates from those in flows over bare channel flow^25,36^. As a result, sediment transport behavior can be particularly altered as the flow evolves through mangrove swamps. The rivers with mangroves contain finer sediments, which carry more organic and nutrient contents than the unvegetated channel^37,38^. Furthermore, sediment transport considerably influences the function, morphology, and turbidity of the river bed and alters fish habitat^39^. Therefore, understanding the mechanism of sediment transport in mangrove swamp requires a detailed comprehension of the hydrodynamics interaction of the mangrove roots in nature, sediments, and flow conditions, as well as a precise predictive model to the erosion process around the prop roots.

One challenge to understanding the onset of sediment transport is the lack of turbulence measurements and detailed flow measurements around the roots close to the mangrove roots. Turbulence is generated as water flows past mangrove roots, and it is expected that the higher porous patch of mangrove models would be associated with a high level of small-scale turbulence^40^. Nevertheless, in principle, turbulence is not always expected to increase with increasing mangrove porosity. In the low porous patch (or high water blockage), water current can become so sluggish that only limited turbulence is generated. As a result, there could be an optimal patch porosity that yields maximum energy dissipation from the flow due to the drag, with a lower erosion attributed to a higher velocity required for incipient motion. Furthermore, there is a question about the presence of eco-geomorphological feedback that mangroves grow roots in an optimal root porosity to promote scour near roots to prevent burial, while concurrently creating low energy regions of sediment deposition farther inside the forest, in that way facilitating the propagation of the mangrove swamp.

Near-bed turbulence plays an important role in controlling the onset of erosion and therefore impacts the sediment distribution by converting large-scale current energy into dissipative wake scale turbulence^41,42^. The onset of sediment transport is related to the velocity fluctuations and skin friction coefficient.

Predictive models that are used for sediment transport over the bare channel do not apply to the vegetated channel because of the effect of vegetation on the velocity profile and turbulence production^43,44^. In the unvegetated channel, the initiation of sediment transport is conventionally related to the bed shear stress ($\tau _b$) which can be estimated by several methods comprising logarithmic law of wall, water surface slope method, and using the empirical relation between turbulent kinetic energy and bed shear stress^45^. To overcome these difficulties various models have been proposed^43,46,47^. However, these methods are not applicable or appropriate to vegetated channels. Yang et al.^43^ proposed a Linear Stress Model (LSM) that defines a viscous layer immediately above the bed, within which the viscous stress decreases linearly with distance from the bed, and leads to a parabolic velocity profile. However, this assumption neglects the turbulent terms which are generated by the roots. Yang et al.^43^ showed that the shear stress on the bare bed cannot be predicted by the linear vertical distribution of turbulent shear stress to the bed because the shear stress profile is not predefined. It is essential to obtain the bed shear stress in the mangrove root region based on measurable parameters such as patch root porosity and flow conditions.

In this work, we used wooden circular cylinders as a simplified mangrove root model that is often used to model rigid emergent vegetation as it provides an appropriate approximation of the root^48,49^. Importantly, the initial growth of the roots often forms circular cylinders with a low number of roots and expand laterally with root size diameter^48^.The roots’ dimension, layout, and surface roughness were scaled according to the measurements, and the white root colors were selected for better visualization. We used a velocity range of 2–12 $\frac{\rm{cm}}{\rm{s}}$ consistant with the field observations of water current within heavily vegetated swamps with peak tidal velocities in shallow water depth^50^. The patch porosity under consideration contains a range of porosity scales and is representative that one would likely encounter in nature as reported by Furukawa et al.^51^ which have the porosity up to 45$\%$.

We used Particle Image Velocimetry (PIV) to investigate the interaction of the boundary layer with the near-bed flow using four different patch porosities. The geometrical parameters of all cases are shown in Table 1. PIV measurement can provide direct and detailed velocity measurements over an entire flume bed ^52^. Measurements were first performed with smooth-wall conditions to establish the baseline flow structure within the unvegetated channel followed by measurements for four different patch porosities. The models were tested to develop quantitative models for prop roots effects on the bed. The parameters affecting the incipient motion were investigated to understand how spatially-averaged velocity derivatives alter in the presence of the simplified mangrove roots. Additionally, since the turbulence generated by the vegetation makes the flow spatially variable, we present a spatially-distributed flow parameter behind the root patch.

## Results and discussion

**Figure 2:**
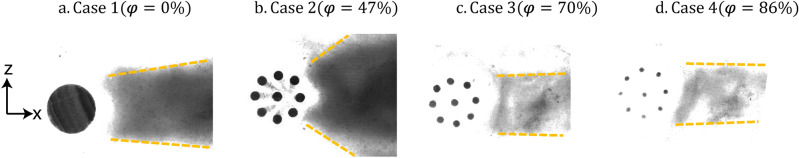
The sediments on the flume bed are indicated by the grayscale image of the sediment trajectories deposited on the flume bed. The flow was from left to right in the image. The black circles indicate the bottom positions of mangrove root-type models emergent in the water. The camera was positioned below the water tunnel in the horizontal plane. The patch with $47\%$ exhibits the minimum erosion in the near-wall mainly due to the lower turbulence and velocity in the near-wall region. The geometrical parameters of all cases are shown in Table 1.

After the onset of the sediment transport, it was observed that some sediments followed the streamwise direction along the flume with no indication of deposition on the bed. However, most sediments tended to deposit after the erosion took place and stayed unmoved. This trend formed a distinctive depositional region based on the area and density of the sediment accumulation. The pattern of sediment erosion posterior to the mangrove root-type models is shown in Fig. 2a–d. For all cases, the sediment deposition region was less pronounced immediately behind the patches, however, after approximately 3 mm behind the patches, we observed the formation of the deposition region. It was also observed that most sediments were eroded for case 3 and 4 which are the models with high porosity (less blockage). For the low porosities (case 1 and 2), a convex pattern of the deposition region was shaped and for the high porosity (case 3 and 4), the rectangular-like region was formed. However, the sediment deposition area for case 2 ranged from 25 to 75 $\text{cm}^2$, the largest area among others, indicating that mangrove root porosity could be related to mitigating the erosion of posterior to the patch even compared to the single solid cylinder (case 1). This signifies that for a fixed root configuration there could be an optimal porosity to mitigate erosion.

**Figure 3:**
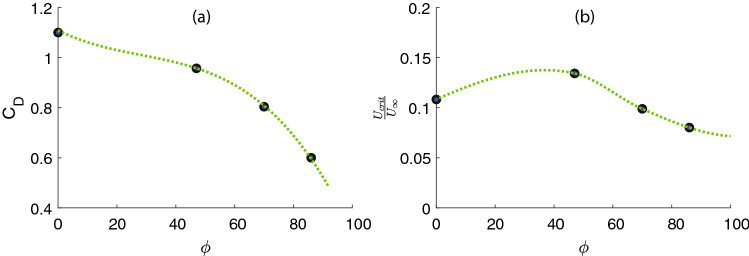
(**a**) The drag coefficient versus patch porosity^25^. (**b**) Critical velocity obtained based on Eq. (1) which indicates initiation of the sediment transport. Erosion occurs if the flow velocity over the sediments is exceeded by the critical velocity that reached the peak at $\phi =47\%$.

**Figure 4:**
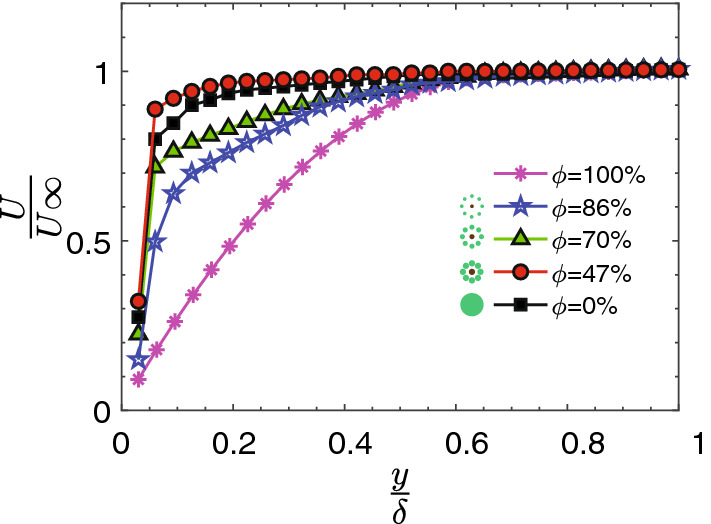
Streamwise velocity profile, *U*(*x*) at various porosity parameter normalized by boundary layer thickness ($\delta$) and upstream velocity ($U_\infty$).

Based on the reduction in the turbulence production due to the mangrove roots, the critical incipient velocity revealed a decreasing trend with porosity except for the second case (Fig. 3a,b), which has an approximate normalized critical velocity of 0.13. Figure 3a shows drag coefficient, based on the patch diameter, that varies with patch porosity^25^. Figure 3b is obtained using $C_f$ that we measured in the experiment and found that at $\phi =47\%$, the critical erosion velocity is much lower than the other cases, indicating a lower possibility of the sediment erosion in the near-bed (Fig. 3b). To understand the phenomenology of this pattern, we considered the near-bed flow structure disturbed by the mangrove root patches. The velocity profiles of the boundary layer were acquired at stream-wise locations for four values of the porosity’s at $U_\infty =2 \, \frac{\rm{cm}}{\rm{s}}$ corresponding to Reynolds number of 2500, based on the patch diameter. In Fig. 4, the normal distance is normalized by the boundary layer thickness ($\delta$) which is defined as the value of *y* at which *U* equals $99\%$ of the free stream velocity ($U_\infty$). For the smooth-wall case ($\phi =100\%$), as the flow approached the bed, the velocity achieved zero value as dictated by the no-slip conditions. Therefore, a velocity gradient was formed in a normal direction to the flow due to the viscosity effect.

The mean velocity profile (Fig. 4) revealed a parabolic trend with the mean profile for fully developed laminar flow for the smooth wall. With increasing the porosity, the mean velocity profile deviated from the laminar characteristics and became progressively flat as the flow transitions from a laminar to a turbulent flow. Specifically, the boundary layer thickness for $\phi =47\%$ is approximately 10% lower than the solid cylinder value and thus this results in larger velocity gradients in the region close to the wall which accounts for increased values of wall shear stress.

**Figure 5:**
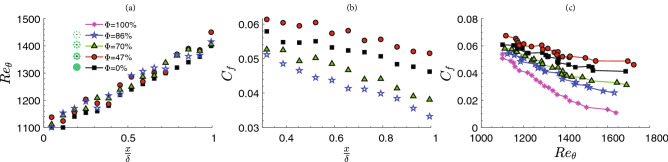
(**a**) Distribution of Reynolds number based on the momentum thickness ($\theta =\int (1-\frac{u}{u_\infty })dy$) in streamwise direction. (**b**) skin friction coefficient with respect to distance from the patch and (**c**) with respect to the Reynolds number.

The spatial distribution of the momentum thickness exhibited a linear change with streamwise distance from the patch (Fig. 5a) with an insignificant porosity effect. The momentum thickness ($\theta =\int (1-\frac{u}{u_\infty })dy$) represents the loss of momentum due to the presence of a wall and increased with the distance from the patch.

However, it remained almost unchanged with respect to porosity. The streamwise distribution of skin friction coefficient, $C_f$, measured for four cases of different porosities (Fig. 5b) indicated that the baseline case (smooth wall) skin friction coefficient ranged from 0.03 to 0.05. However, with the presence of mangrove roots, the average skin friction coefficient $C_f$ decreased with porosity. It implies that for a constant loss in the fluid momentum, the skin friction factor opposing bed drag is high for the $\phi =47\%$. In addition, it can be observed that the skin coefficient factor for a constant momentum thickness is the largest for case 2 (Fig. 5c).

**Figure 6:**
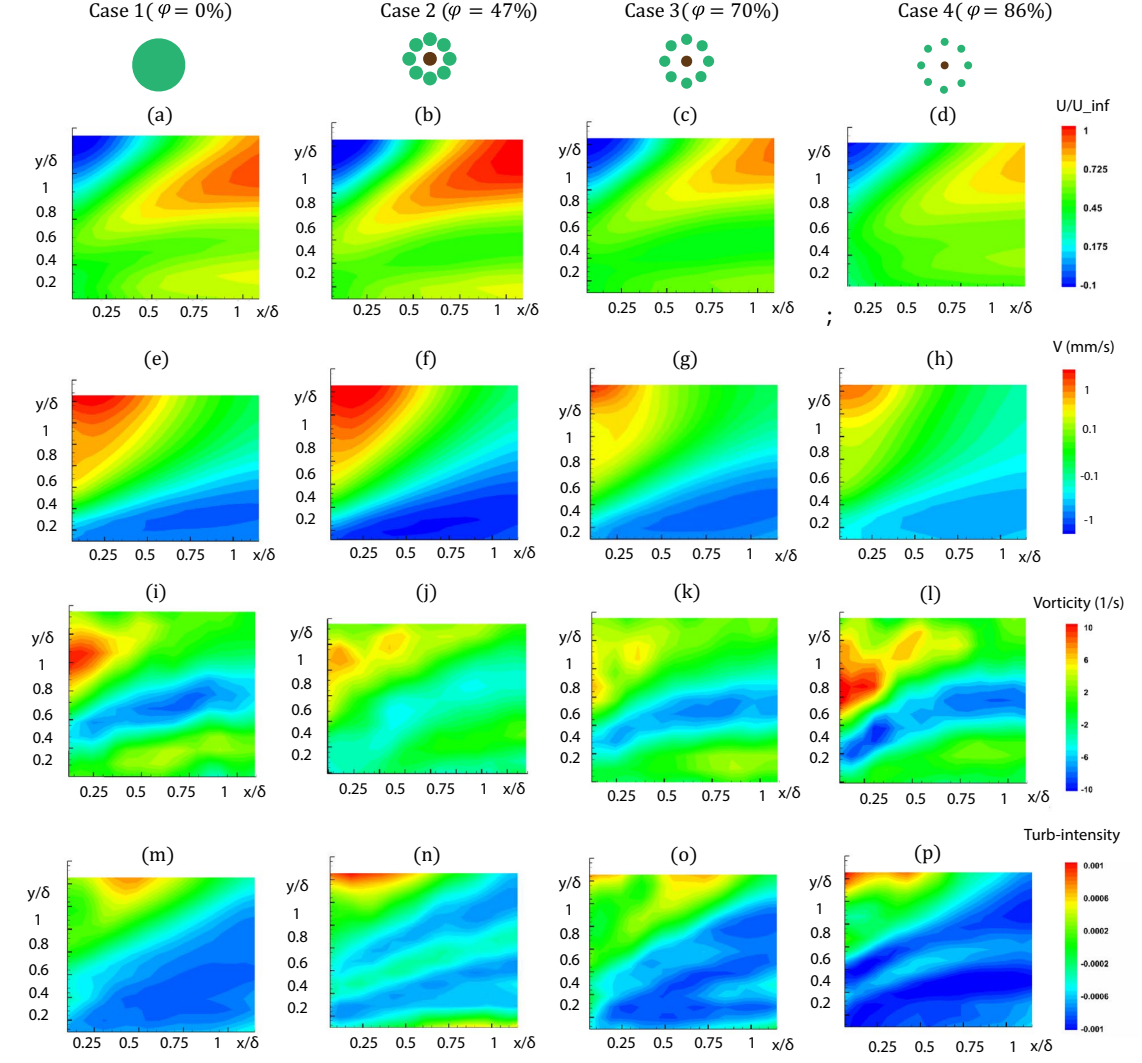
Time-average flow fields downstream of the patches for different porosities. Left column: case 1, $\phi _1=0\%$; Second column: case 2, $\phi _2=47\%$; and second from right column: case 3, $\phi _3=70\%$; Right column: case 4, $\phi _4=86\%$; (**a**)–(**d**) Streamwise velocity; (**e**)–(**h**) contour of velocity in the Y direction. (**i**)–(**l**) contour of vorticity; (**m**)–(**p**) contour of turbulence intensity (TI). All results are at Re = 2500 with time step t = 0.008 s and form time period of T = 8 s.

To examine the distribution of parameters in the flow structure, the spatial variation of the streamwise velocity (*U*), spanwise velocity (*V*), vorticity ($\omega$), and turbulence intensity are shown in Fig. 6 for different porosities. Locally, there is a small area with negative streamwise velocity (Fig. 6a–d) in blue immediately downstream of the patches. However, by decreasing the porosity, the positive streamwise region increases. It is noted that for the $\phi =47\%$ the elevated streamwise and spanwise velocity (Fig. 6e,f) is mainly due to the augmented flow constraints by the neighboring cylinders in the patch with $\phi =47\%$ porosity. The difference between the maximum and minimum local streamwise and spanwise velocities can also be seen to decrease significantly with patch porosity (Fig. 6g,h). This decrease in velocity change has also been observed in flow through random cylinder configurations^53^.

Therefore, an area with positive vorticity ($\omega$) emerged downstream of the patch due to the recirculation in the wake region. As the patch porosity decreases, the flow that is channeled between cylinders follows an increasingly tortuous path. Vorticity change in the middle of the wall region is relatively small because the fluid particles can acquire rotation only by viscous diffusion which is the low value at the end of the buffer layer. During the time fluid traveled downstream of the patch, it was observed that the sediments diffused only a small distance away from the boundary layer.

Similarly, the fluctuation of streamwise velocity indicated by turbulence intensity (Fig. 6m–p) was around 0.001 for the least porous patch and decayed with porosity increase. The patch with $\phi =47\%$ exhibited the nearly zero value for the vorticity and turbulence intensity compared to the impermeable cylinder and other patches (Fig. 6j–n). This deficiency in velocity fluctuation elevated the possibility of sediment deposition in the near-wall region for case 2.

**Figure 7:**
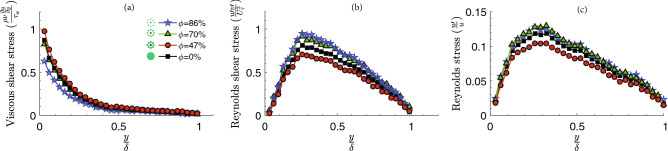
Spatially-averaged of flow parameters changing immediately behind the models. (**a**) Viscous shear stress changes with distance from the bed normalized by the boundary layer thickness ($\updelta$). (**b**) Variations of Reynolds shear stress normalized by shear velocity ($U_\tau$). (**c**) Reynolds stress variations from the bed. It was observed that the total shear stress was constant across the layer and equal to the wall shear stress ($\tau _w$).

Even though the viscous shear stress has a low value for the high porous patch, it is relatively unchanged with porosity increase in farther distance from the patch (Fig. 7a). Importantly, for case 2, sediments most likely experienced high resistance forces as the normalized viscous shear stress was around one, which contributes to a lower erosion in the near-wall region. Figure 7b presents the profile of Reynolds Shear Stress (RSS $=\rho U ^{\prime} V^{\prime}$) for the root patches at Re=2500. Furthermore, at a fixed $\frac{y}{\delta }$, excellent collapse of the RSS profile for all cases is noted, where the profile exhibits a linear region from the centerline to the peak location. This linear behavior confirms the characteristics of transitional and turbulent flow and shows the dominance of turbulent (inertial) effect over viscous stress. As the wall is approached, deviation from the patch was noted for the rough patches as the magnitude of the peak in the RSS increases with increasing porosity, coupled with a slight shift in the peak location close to the wall. Figure 7c indicates that the streamwise velocity fluctuations were sufficient for all cases ($\frac{U ^{\prime} }{U}>10\%$) to grow the instability in the near-wall region and Tollmien–Schlichting (T-S) waves emerge in the boundary layer . The T-S waves are formed when the disturbance interacts with the roughness in a process also known as receptivity^54^. These waves are gradually augmented as they move downstream until they may eventually grow large enough that nonlinearities conquest and the flow transitions to turbulence.

The reduction in the velocity fluctuations can be elucidated based on the vorticity budget behind the patch for case 2. To understand the reason for this phenomenon, we refer the spacial distribution of vorticity posterior to the patch (Fig. 6i–l) which exhibits nearly zero value vorticity in the near wall region. This is mainly due to the sink of the vorticity as can be elaborated using the vorticity evolution equation for a two dimensional, viscous incompressible flows rewritten as:

1 $$\begin{aligned} \frac{D\omega }{Dt}=\nu \nabla ^2 \omega =\underbrace{\frac{1}{Re}\left[ \nabla \times \left( \frac{1}{\rho }\nabla \cdot \tau \right) \right] }_{viscous(\omega _s)} \end{aligned}$$

The key parameter is the viscous-term ($\omega _s$) with important effects owing to variation in the coefficient of viscosity in the boundary layer^47,54^. The spatially averaged values of different terms at each stream-wise location are plotted for all cases. The viscous term can be further divided into two components:

2 $$\begin{aligned} \omega _s=\frac{1}{Re}\underbrace{\left[ \nabla \times \frac{1}{\rho } \left( \frac{\partial \phi _a}{\partial x}i+\frac{\partial \phi _b}{\partial y}j\right) \right] }_{\omega _{s1}}+\frac{1}{Re}\underbrace{\left[ \nabla \times \frac{1}{\rho }\left( \frac{\partial \phi _c}{\partial y}i+\frac{\partial \phi _c}{\partial x}j\right) \right] }_{\omega _{s2}} \end{aligned}$$

where for a 2-D, incompressible flow in the boundary layer $\phi _a=\phi _b=0$ and $\phi _c$ is calculated as $\phi _c=\mu (\frac{\partial v}{\partial x}+\frac{\partial u}{\partial y})$.

**Figure 8:**
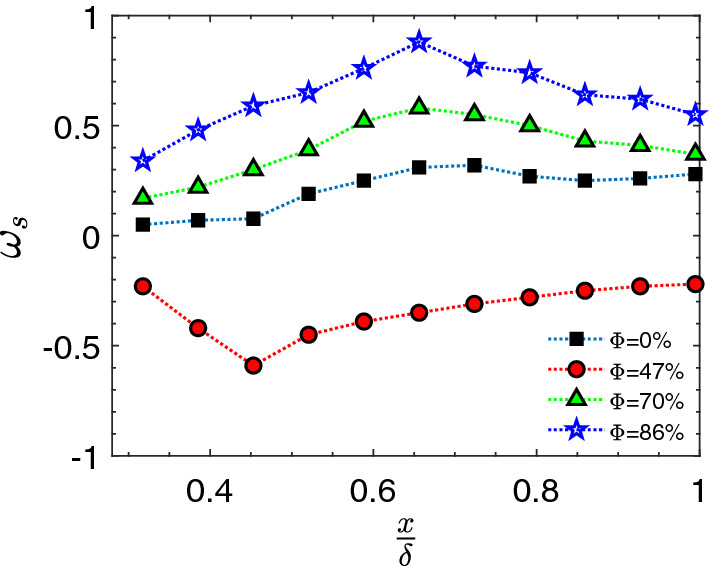
The variation of the viscous term in the vorticity evolution equation act as source and sink of the vorticity that causes the lift augmentation around the sediments.

In fact, $\omega _{s2}$ acts as sources or sinks of vorticity in the near-bed for the controlled case depending on its sign. It also implies the relationship between vorticity generation in the boundary layer and velocity gradient which account for shear stress at the wall. Figure 8 shows that the magnitude and distribution of $\omega _{s2}$ and verifies that the reduction in the vorticity is dependent on the presence of the root patch. Note that for case 2 ($\phi =47\%), \omega _{s2}$ is negative at the beginning and remains almost negative downstream of the patch. It confirms the reduction in the decay of the vorticity with the presence of mangrove root models consistent with the field experiment by Furukawa et al.^51^ and Wolanski^55^ that observed sediment trapping by mangroves. This reduction in the vorticity makes the boundary layer quiescent with lower rotation in the flow, consequently, the greater velocity gradient in the near-wall region. Therefore, the boundary layer thickness $\frac{y}{\delta}$ has less than 0.1 value for $\phi =47\%$ (Fig. 4).

It is also important to note that for the higher porosities, the vorticity is higher and thus the velocity gradient in the near-wall is lower compared to the lower porosity. Therefore, the boundary layer is similar to the unvegetated channel with a low near-wall velocity gradient and consequently low $C_f$ values (Fig. 5b,c).

**Figure 9:**
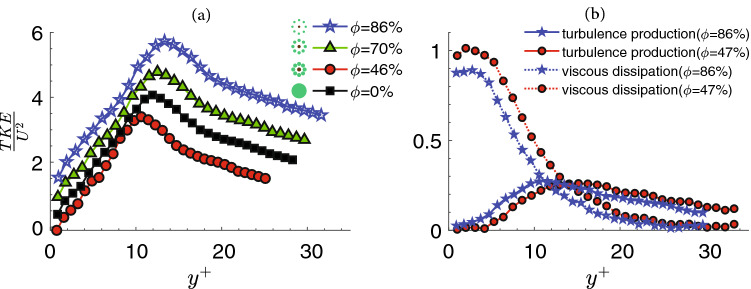
Variations of spatially-averaged of flow parameters with non-dimensional wall-normal distance behind the models. (**a**) Turbulent kinetic energy budget. (**b**) Turbulence production versus viscous dissipation.

Turbulent Kinetic Energy (TKE) rises steadily from almost zero at the wall to a peak value at $y^+\approx 12$ and then declines through the upper part of the buffer region as it proceeds towards the downstream. The distribution of TKE downstream of the mangrove-root models is characterized by a noticeable peak (Fig. 9a) suggesting the turbulent region behind case 2 is less than $y^+<10$. It is mainly due to the local viscous dissipation that significantly exceeds the production of kinetic energy in the near-wall region (Fig. 9b). Thus, there is strong cross-stream diffusion of energy both towards the wall and towards the core flow. The energy balance of the mean motion can be rewritten as:

3 $$\begin{aligned} \frac{\mathrm{d}u^+}{\mathrm{d}y^+} = \left (\frac{\mathrm{d}u^+}{\mathrm{d}y^+} \right)^2+\tau _t^+\frac{\mathrm{d}u^+}{\mathrm{d}y^+} \qquad \end{aligned}$$

Where the first term on the left-hand side is related to the energy supply, the second term is viscous dissipation, transformed into internal energy and the third term is turbulence production which is used to generate turbulent fluctuations energy. This term would eventually transform into internal energy. The turbulence has a maxim of 0.25 at $y^+=10.6$ (Fig. 9b).

At this distance from the wall, the viscous dissipation and turbulence production are equal. For $y^+<10.6$ the viscous dissipation dominates whereas for $y^+>10.6$ the turbulence production dominates. This is mainly due to the Reynolds stress works against the mean velocity gradient to remove energy from the mean flow, just as the viscous stress works against the velocity gradients. However, the energy removed by the viscous stress is directly dissipated, reappearing as heat, but the action of the Reynolds stress provides energy for turbulence fluctuations. The loss of mean flow energy to turbulence is large compared with the viscous dissipation. Consequently, it implies that for case 2 the turbulence production is not sufficient to exceed the viscous dissipation and discourage the initiation of sediment transport.

**Figure 10:**
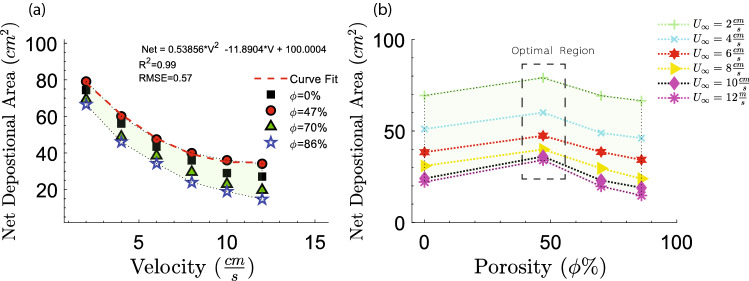
Net depositional area behind the patch versus upstream water velocity (**a**) and patch porosity (**b**) the optimal porosity is $\phi =47\%$ and the curve fit shows it variations with the velocity. The green area indicates the region between high and low net depositional areas.

We considered the change in net sediment deposition integrated over a squared area with length scale 4D (approximately 10 cm) centered directly behind the patch. Net deposition was defined as the total deposition behind the squared area with the presence of the root models minus the total deposition behind the squared area in the free stream (bare channel). Even though Fig. 10b reveals the optimal porosity for velocity range of $2{-}12 \, \frac{\rm{cm}}{\rm{s}}$, the net sediment deposition decreased quadratically with respect to the upstream velocity indicating an insignificant dependence on the mangrove roots’ patch porosity in low velocity (Fig. 10a). In contrast, for the higher velocities $((U>6 \; \frac{\rm{cm}}{\rm{s}}))$, the net sediment deposition difference between high porous patch ($\phi =86\%$) and the optimal porosity patch is more pronounced. This can be elucidated based on the turbulence generated in the boundary layer for the wake of the mangrove roots impacted sediment deposition and erosion for the optimal patch. First, low turbulent motions deposited more individual sediment particles in the boundary, and second, low wake turbulence diminished vertical diffusivity and thus increased the ability of a flow to keep sediment in suspension, facilitating sediment transport and therefore increasing the sediment deposition.

## Discussion

We presented necessary information to address the onset of sediment transport that can provide useful insights into the current challenges to the global restoration community and bring about opportunities for interdisciplinary collaboration with the ecological engineering community. Accurate projection of hydrodynamic erosion and the essential amount of mangrove species has been a challenge for managers and restoration practitioners to envisage a successful component of project designs. This necessitates acquiring two critical pieces of information: (1) Characterizing the near-bed boundary layer of the mangrove roots and their effect on the mangrove root erosion. (2) Quantitative understanding of mangrove root erosion and the habitat requirements based on the optimal porosity.

This paper aimed at quantifying the hydrodynamic conditions that affected the onset of sediment transport will address the first informational need for global restoration communities with mangrove habitats. Characterizing the hydrodynamics of mangrove-like structures can elucidate the primary mechanisms for its resilience and by which mangrove roots can withstand high-energy fluid conditions. For example, we observed that most sediments are eroded for the case with high porosity (less blockage), and the sediment deposition region for the low porous patch ($\phi =47\%$) had the maximum area (ranged from $25\, \text{cm}^2$ to $75\, \text{cm}^2$) among others (Fig. 2) signifying an optimal porosity to mitigate erosion for a fixed root configuration. This information has the potential to improve future coastal infrastructure design with bio-mimetic mangrove-like structures.

For the second piece of information, hydrodynamic of mangrove roots and the proposed predictive model for the onset of erosion, as well as the critical velocity, porosity range, the near-bed boundary layer parameters reported here for mangrove roots, can be applied to support restoration decision making. The optimal configuration porosity range and the critical velocity presented in this study can provide useful guidance for coastal managers restoring estuarine mangrove forests or planting mangroves as part of living shoreline stabilization. As an example, by understanding the range of porosity under a given set of hydrodynamic conditions (Fig. 3) coastal managers can weigh the effectiveness of energy abortion in terms of drag coefficient (Fig. 3a) and benefits of lower erosion at higher porosity (Fig. 3b) and use additional supplementary design components to do the trade-off between them.

Additionally, this study implies that optimal porosity design of shoreline may add habitat flexibility to sites that are on the borderline of mangrove habitat suitability. This optimal porosity would affect increase the critical velocity at which the sediment transport initiates. The increment in the critical velocity has biological importance as it could potentially increase nutrients around the roots, increase energy dissipation to withstand high flow speeds, control changes substrate bottom to facilitate the propagation of mangrove swamp. Thus, the roots which do not exceed the critical porosity for maximum energy dissipation may have adaptive benefits, for example, the ability to tolerate brackish waters, in depositional environments. Increasing the mangrove species through pre-restoration grading can potentially increase the likelihood of decreasing erosion success, with a higher energy dissipation that increases the resistance of mangrove roots to the energy in tidal flows. It should be noted that even though mangroves may have been traditionally present at a site before deforestation, managers should not rush to replant. Anthropogenic modifications to the offshore flow conditions such as bathymetry, water levels, with subsequent wake dynamics may substantially affect the erosion initiation.

The proposed critical velocity for erosion initiation is suitable for locations where the tide and geomorphology of the coastal lagoons provide a minimum tidal exchange with water current up to $12 \; \frac{\rm{cm}}{\rm{s}}$. In particular, for the Gulf of Mexico where there is neap tides with a limited tidal amplitude as well as in Caribbean Sea, the coast of Thailand, intercoastal zones in India, and other low tide zones around the world. Even though the arrangement of the cylinders in the patch affects the flow structure in a patch of cylinders, it is mainly a function of porosity which is a geometrical parameter that includes the solidity of the individual cylinder inside of the patch over a volume consistent with fieldwork performed by Mazda et al.^56^ In addition, a drag force change due to a change in the arrangement of the cylinders will not be captured in a drag coefficient based on the patch diameter (D), however, it could be captured based on the effective diameter^25^.

Although the root models were based on the structure of an isolated patch of Rhizophora mangrove prop roots, our results could have implications for investigations of other fluid challenges, including flow through either porous object, vegetation, or an array of objects. Furthermore, understanding the hydrodynamics and scaling of this problem could also contribute to the design and development of a bio-inspired mangrove-like system for coastal protection globally especially in the (sub)tropical regions with the possibility of mangrove growth. While this is the first study to quantify the optimal mangrove root hydrodynamic with a predictive model, we suggest that observational studies should be performed in a large number of sample sites to predict the probability of mangrove erosion based on the correlation between hydrodynamics and observed mangroves distributions. The field studies wide spatiotemporal parameters may extend the results of the current research to successfully predict mangrove erosion outcomes on estuarine shorelines.

## Conclusion

We presented simplified mangrove root type models with different porosities to investigate the impact of porosity on the incipient motion of the flow which is critical to the evolution of shorelines, delta, and lands. It is observed that most sediments are eroded for the case with high porosity (less blockage) and the sediment deposition region for the low porous patch had the maximum area among others signifying an optimal porosity to mitigate erosion for a fixed root configuration. We performed PIV measurements to investigate the impact of the mangrove root-type models on the near-bed boundary layer disturbed by mangrove roots that affected the onset of sediment transport. High-resolution time-resolved velocity field and its derivatives were obtained in the streamwise wall-normal ($\mathrm {x-y}$) plane at midplane for the flow through the channel fitted with the mangrove-root patch. We proposed a predictive model for the critical velocity of simplified mangrove roots incorporating geometrical and hydrodynamic parameters. We found that $\phi =47\%$ is an optimum porosity for sediment erosion in the near-bed region. This was mainly due to the nearly zero turbulence and vorticity due to the act of sink term in the vorticity evolution equation. Further evaluation of the boundary layer attained by PIV evinced that viscous dissipation dominates the turbulence production for the optimum porosity. Our work contributes to fill a gap in understanding the near-bed flow and step forward accurate prediction of sediment transport in beds which contributes to shaping the beds in nature.

## Methods

### Theoretical considerations for the critical velocity

**Figure 11:**
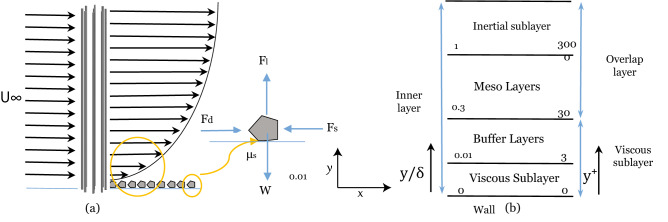
(**a**) Velocity profile over the bed with the presence of a mangrove root model that produces large roughness as the boundary layer is formed with thickness $\delta$; The interacting forces on arbitrary sediment are drag and lift forces that encourage incipient motion and sediment weight force that resist the motion. Sediment transport begins when the combined lift and drag forces produced by the fluids become large enough to counteract the gravity and frictional forces that hold the sediments in place. Since the mangrove models generate a turbulent boundary layer, a turbulence-based model can predict the incipient motion. (**b**) The structure of a turbulent boundary layer for smooth surfaces based on wall-normal distance ($y^+=\frac{yU_\tau }{\nu }$).

Figure 11a shows the velocity profile posterior to the patch root models facing the upstream velocity of $U_\infty$. The acting force on arbitrary sediment at the wall is displayed in Fig. 11a. $F_L$ and $F_D$ are lift and drag forces on the sediments, respectively. $F_s$ is the resistance force and *W* is the weight of the sediments. Prior to the sediment transport initiation, forces are balanced, however, by increasing the velocity, the lift and drag force overcomes the friction force and particle weight initiating the sediment transport. These forces are difficult to measure since direct force measurement on individual particles is a significant challenge in practice. Therefore, we employed a turbulence-based prediction model for initiating the sediment transport with the presence of the mangrove roots models.

The incipient motion of sediments is associated with the turbulent flow eddies and velocity fluctuations in the near-bed, that produce sufficient lift and drag force to disrupt the sediments^57,58^. Thus, we used $k_t$ to determine turbulent kinetic energy. In an unvegetated channel $k_t=CU^2$, where *C* denotes a constant dependent on the skin friction coefficient. In a vegetated channel, $k_t$ depends on the turbulence generated both by the bed and the roots and the mutual effect of the bed and roots. For $Re_d>120$ (*d* is the root diameter), the root-scale turbulence is $k_r=1.2[C_D\frac{1-\phi }{\phi \frac{\pi }{2}}]^\frac{2}{3} U^2$, for $\phi \ge 85$ and $k_r=0.77[C_D\frac{L}{d}\frac{1-\phi }{\phi \frac{\pi }{2}}]^\frac{2}{3}U^2$ for $\phi \le 85$, where, $C_D$ is root drag coefficient and $\phi$ is root patch porosity^59^. Assuming that the total near-bed turbulence ($k_t$) is the sum of the root ($k_r$) and bed $k_b$, the total near-bed patch can be estimated as $k_t=k_r+C_fU^2$. By assuming the onset of sediment motion is controlled by $k_t$ (i.e $k_t=k_{t\,Critical}$), we proposed the critical velocity can be predicted by:

4 $$\begin{aligned} C_{f\,smooth}U_\infty ^2 = {\left\{ \begin{array}{ll} C_f U_{critical}^2+1.2\left[ C_D\frac{1-\phi }{\phi \frac{\pi }{2}}\right] ^\frac{2}{3} U_{critical}^2+\frac{T \cdot K \cdot E}{U_\infty ^2}U_{critical}^2 \qquad \qquad \hbox { for}\ \phi \ge 85\%, \\ C_fU_{critical}^2+0.77\left[ C_D\frac{L}{d}\frac{1-\phi }{\phi \frac{\pi }{2}}\right] ^\frac{2}{3} U_{critical}^2+\frac{T \cdot K \cdot E}{U_\infty ^2}U_{critical}^2\qquad \quad \hbox { for}\ \phi \le 85\% \end{array}\right. } \end{aligned}$$

where, $U_{critical}$ is the velocity which indicates the onset of sediment transport, $C_f$ is skin friction coefficient of the bed, $C_D$ is the drag coefficient of the patch that we measured^60^, *T*.*K*.*E* is the near-bed turbulent kinetic energy due to the mutual effect of the roots and bed, and $\frac{L}{d}$ is the spacing ratio that is equivalent to $\frac{L}{d}=\frac{1-\sqrt{\frac{1-\phi }{N}}}{2}$, where *N* is the number of roots. The fluctuating fluid forces are connected to the near bed-turbulent kinetic energy ($k_t$), therefore, $k_t$ can be used as a predictor of the initiation of sediment motion^46^. Equation (4) implies that the skin friction coefficient in near-bed flow structure causes the sediment’s resistance to erosion, governed by the presence of the roots, contributes to the larger sediment deposition region. An increase in the critical velocity where mangrove roots are present leads to extra sediment deposition. This indicates that, in addition to the vegetation-induced channel erosion, enhanced sediment deposition also plays an important role in the formation of the channel network.

The interaction of the fluid flow and smooth channel generates a boundary layer where the fluid velocity is zero at the interface with the channel and the velocity increases away from the wall until it reaches the free stream velocity ($U_\infty$). The structure of the turbulent boundary layer for a smooth surface is considered based on wall-normal distance ($y^+=(U_\tau y)/\nu$, where $U_\tau$ is shear velocity and $\nu$ is the kinematic viscosity of water), this normalization is undertaken to magnify the displacement across the boundary layer (Fig. 11b). The linear sublayer region extends from the near-wall up to $y^+$ = 3 where the viscous forces characterize the flow. In the buffer layer, the combination of viscous stress and Reynolds stress dominates the region from $3<y^+<30$. The mesolayer plays a critical role in energy transfer. In this layer which extends from $30<y^+<300$ turbulence behaves like low Reynolds number turbulence regardless of the Reynolds number range in the outer layer^61^. This layer contains the effects of viscosity for all turbulence scales even at high Reynolds number^62^. For the low Reynolds number, as in the present work, the mesolyaer constructs about $25\%$ of the outer boundary layer. In the inertial sublayer ($y^+>300$), a constant stress layer exists for the flow. Inertial sublayer forms at about $Re_\theta =10000$^63^ (where, $\theta$ is the momentum thickness of the boundary layer), that was not observed in the present work.

The mangrove root models are expected to disrupt the near-wall region depending on the roughness, configurations, and patch porosity. The modification of the boundary layer is highly important because it shows the reduction of viscous effects on the near-wall region where the initiation of sediment transport is connected to the bed shear stress. Widdows^46^suggested $\tau =0.19 k_t$ ($k_t$ is turbulent kinetic energy) to estimate the mean bed stress from the measured turbulent kinetic energy. However, this relation assumes that turbulence production is merely connected to bed stress, which is not true in vegetated systems. In a vegetated channel, vegetation plays a critical role in turbulence production^43,44^. The total bed shear stress equation in the boundary layer along the flow is:

5 $$\begin{aligned} \tau =\underbrace{\rho \nu \frac{\partial u}{\partial y}}_\text {Viscous Shear Stress}-\underbrace{\rho \langle u'v'\rangle }_\text {Reynolds Shear Stress} \end{aligned}$$

where, $\tau$ is total shear stress, and $u'$and $v'$ represent the velocity fluctuations. At the wall, $\langle u'v'\rangle =0$ and $\rho \nu \frac{\partial u}{\partial y}$ is wall shear stress ($\tau _w$). Thus, in the near-wall region, the wall shear stress ($\tau _w$) is composed of viscous stress $\tau _w=\rho \nu \frac{\partial u}{\partial y}$ and form drag ($\tau _{FD}=-\rho \langle u'v'\rangle$). The skin friction coefficient is determined by using wall shear stress and friction velocity ($U_\tau =\sqrt{\frac{\tau _w}{\rho }}$):

6 $$\begin{aligned} \frac{C_f}{2}=\frac{\tau _{w}}{\rho U_{\infty } ^{2}} =\left( \frac{U_\tau }{U_\infty }\right) ^2 \end{aligned}$$

where, $U_\tau$ is the friction velocity and $U_\infty$ is the velocity in the centerline of the flume. The roughness due to the mangrove roots models affect the near-wall region.

### Experimental apparatus and optical system

**Figure 12:**
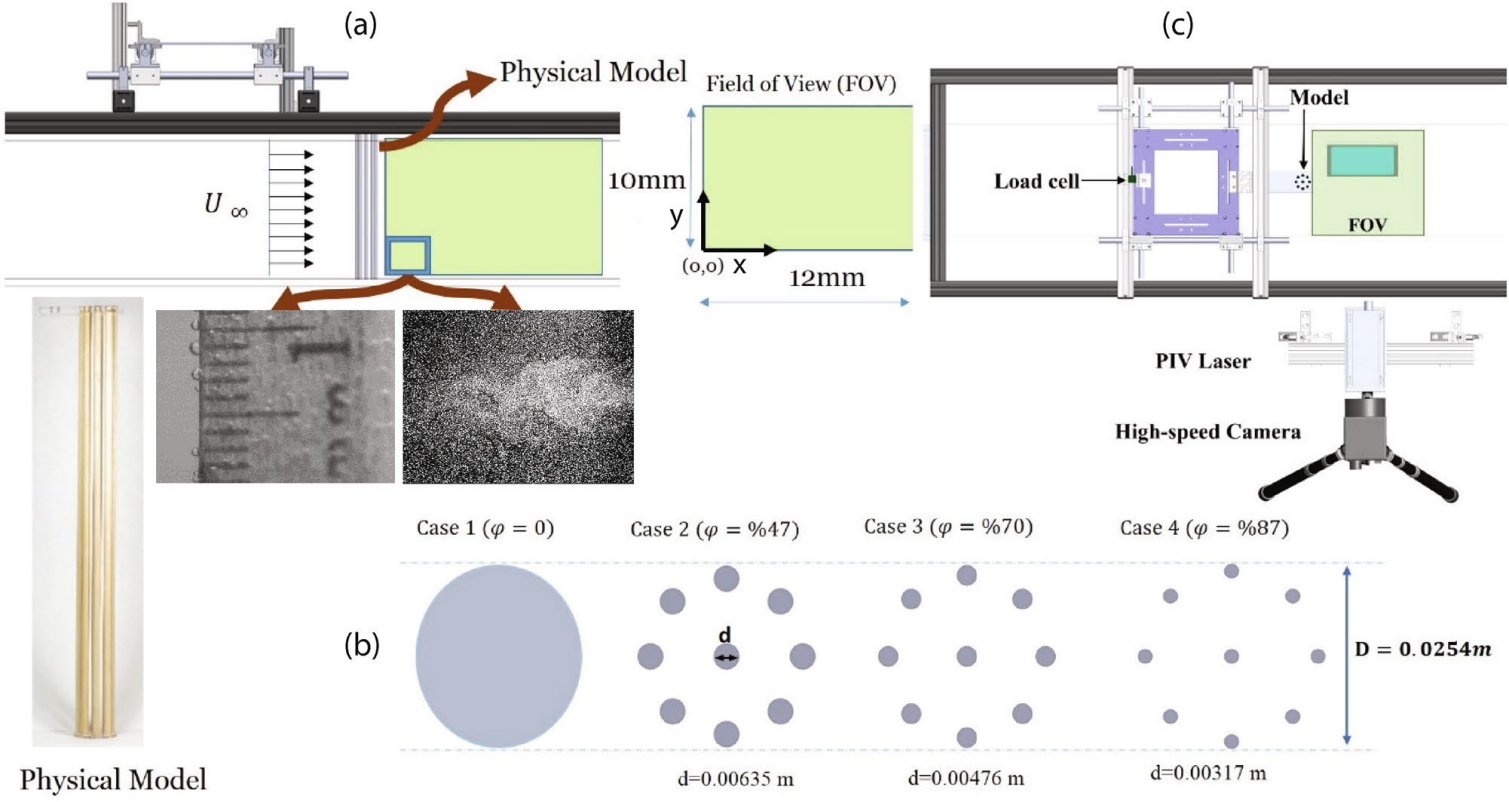
Schematic of the experimental setup and field of view (FOV) located in the mid-depth. (**a**) Side view of the experimental setup. (**b**) a picture of one physical model along with path configurations for different porosities. (**c**) top view of the experimental setup with simplified mangrove root models. The closed-loop water tunnel cross-section is 25 cm $\times$ 25 cm with the blockage ratio of 10%. The setup includes a recirculating flume, an air-bearing system to mount the models, a high-speed camera, and PIV laser. A 45$^\circ$ mirror was used to have a bottom-up view of the flow field.

The experiments were performed in a recirculating water flume with 2 m long and 0.25 m wide and 0.25 m height with a contraction ratio of 3.5:1 moving into the test section with a free stream turbulence level of about $0.1\%$. A schematic of the experimental apparatus is shown in Fig. 12a–c. We conducted the experiments in the flume without vegetation (i.e. bare channel) and with mangrove root models of different porosity. We used wooden circular cylinders with in-line pattern (Fig. 12b) representing simplified mangrove roots that were vertically mounted and extended through the water column. The cylinder tops were fixed to plexiglass attached to the experimental setup and the bottoms were fixed to the bed flume. The distance from the water channel inlet to the models was 1m. For each case, experiments were run for five different upstream velocities from 2 to 12 cm/s and showed that flow patterns were similar. Hence, due to the flow regime similarity in this range of velocity, we selected the velocity of 2 cm/s. The depth of the submerged portion of the cylinders was 22 cm. The cylinders were assembled with a tight fit through the end plates and with no clearances to prevent any deflections at either end and to minimize the three-dimensional flow effects (Fig. 12a). Additionally, we performed experiments in the $x-z$ plane at different *x* locations until the flow became periodic behind the path models to ensure two-dimensional measurements accurately reveal the flow feature. The blockage ratio in the test section, defined as the patch diameter divided by channel width, was around $10\%$. The flow rate was adjusted by a centrifugal pump and ABB frequency controller. The upstream flow velocity was adjusted from 2 cm/s and the corresponding Reynolds number of 2500 based on patch diameter. The quality of the PIV data was assessed by an analysis of some of the statistical results such as the mean velocity profile, the root mean square and the probability density function of the velocity fluctuations.

We used Particle Image Velocimetry (PIV) system in the middle of the flume to measure the velocity flow field. Water was seeded with hollowed-glass spherical particles with a diameter of 0.5 mm consistent with the muddy environment where mangroves usually grow. The flow was continuously seeded to attain consistency in data rates. The particles were illuminated with a continuous 5-W laser (continuous wattage lasers PIV01251) with a 532 nm wavelength. The motion of the particles was recorded using a high-speed camera (Photron Fastcam Mini UX50) with a resolution of $1280\times 1024$ pixels at 250 frames per second (fps) and a shutter speed of $\frac{1}{1450}$ s. Our previous PIV measurement using the same models revealed that a measurement of 10 mm is representative of the boundary layer (Fig. 12a). The image size was $10\, \text{mm} \times 12 \, \text{mm}$ with an interrogation area of $32\times 32$ pixels. Thus, for each image, the flow field was resolved with 40 and 32 points in *x* and *z* directions, respectively. The data collection was for 1000 paired images. For all cases, the water flume worked continuously to ensure that the flow condition remained identical throughout each experiment. The closest measurement relative to the flume bed was performed at $y^+=3$. Besides, sand sizes ($ds\approx 0.6\, \text{mm}$) were used in the water tunnel running for 3 hours per run and the digital camera was placed approximately 0.1 m below the water tunnel to capture the sediment transport motion. The accumulation of the sand was recorded at 50 frames per second with a 35 mm fixed focal length lens.

**Table 1:** Experiment cases with geometrical parameters. *a* is frontal area per unit volume and is defined as $a=\frac{1}{v} {\sum _{n=1}^{\infty } A_i}$ where $A_i$ is a frontal area of individual cylinders and *N* is the number of cylinders and porosity is calculated by $\phi =1-N(\frac{d}{D})^2$.

Case no	Frontal area (a) ($\text{m}^{-1}$)	Patch diameter (*D*) (mm)	*aD*	Root diameter (*d*) (mm)	Porosity ($\phi \%$)
1	N/A	26	N/A	N/A	0
2	107.6	26	2.8	6.35	47.1
3	80.7	26	2.1	4.76	70.4
4	53.82	26	1.4	3.17	86.5

Porosity is the complement of solidity which is the ratio between volume occupied by the roots to the volume occupied by water for confined space below the water surface^64^.

### Root coring measurements

We selected three sites of dense prop roots using $2\; \text{m}^2$ quadrat on each side of the island (Fig. 1a). We carefully selected the sites that were located far away from trees to focus only on clusters of upright root regions within both the seaward and the forest interior. We performed sequential root coring at low tide in February (winter), May (spring), August (summer), and November (autumn) of 2019 the intertidal zone in Lake Worth Lagoon, Florida (Fig. 1a). At each sampling time, four soil cores were taken from each plot with a steel corer of 6 cm in diameter and 0.5 m in depth. Each soil core was separated into four segments (0–10 cm, 10–20 cm, 20–30 cm, and 30–40 cm). Four core segments of the same soil depth from each plot were pooled as a composite sample for subsequent root separation. Then, the average of root diameters for soil core segment were considered for the blow-ground root. Similarly, the average of root diameters for above-ground segment were scaled to standard lab size cylinder as a model used in our setup. According to the root size measurements, the average above-ground root sizes were approximately 9.51 mm, 14.28 mm, and 19.05 mm that were scaled to lab size cylinder diameter of 3.17 mm, 4.76 mm, and 6.35 mm, respectively. Fine root productivity did not vary significantly with increasing porosity of a cluster of prop roots in the soil.
